# Initial Effects of Dynamic Tape on Foot Arch Height Under Cyclic Loading: A Pilot Study Among University Students

**DOI:** 10.3390/sports13050138

**Published:** 2025-04-30

**Authors:** Ting-Chen Chang, Po-Cheng Cheng, Yu-Hsuan Chung, Chih-Wei Chang, Yen-Nien Chen, Chia-Jung Chang

**Affiliations:** 1 Department of Physical Therapy, Asia University, Taichung 413, Taiwan; ta082277@gmail.com; 2 Department of Orthopedics, Chang Bing Show Chwan Memorial Hospital, Changhua 505, Taiwan; mikemikemike30@hotmail.com; 3 Department of Orthopedics, Show Chwan Memorial Hospital, Changhua 500, Taiwan; supersam9101005@gmail.com; 4 Doctoral Program in Translation Medicine, College of Life Sciences, National Chung Hsing University, Taichung 402, Taiwan; 5 Rong Hsin Translational Medicine Research Center, National Chung Hsing University, Taichung 402, Taiwan; 6 Department of Orthopedics, College of Medicine, National Cheng Kung University, Tainan 701, Taiwan; u7901064@yahoo.com.tw; 7 Department of Orthopedics, National Cheng Kung University Hospital, College of Medicine, National Cheng Kung University, Tainan 701, Taiwan; 8 Department of Dermatology, College of Medicine, National Cheng Kung University, Tainan 701, Taiwan

**Keywords:** dynamic tape, foot arch height, arch index, cyclic loading

## Abstract

Background: Dynamic tape is one of the options for supporting the foot arch in the management of arch-related disorders. However, its mechanical effects on the foot arch remain unclear, particularly under cyclic loading. This study aims to investigate the initial effects of dynamic taping on maintaining foot arch height under cyclic loading among university students. Methods: Thirty-three asymptomatic participants were enrolled in this study. The dynamic tape was applied to the foot with the lower arch to provide support, and the other foot remained untaped as a control. The tape was applied without pre-tension and simply laid straight. Changes in bilateral foot arch height and index were measured using a commercial foot sole morphology assessment device and compared after 6 and 12 min of walking. Results: The arch height did not decrease significantly after walking for 6 or 12 min in either the taped or untaped foot. However, the arch index of the taped foot increased significantly (from 0.258 ± 0.086 to 0.273 ± 0.085) after 12 min of walking, whereas no significant change was observed in the untaped foot. Conclusions: This study is the first to evaluate the initial effect of dynamic tape applied without pre-tension on foot arch support by directly measuring sole morphology using a pin-array impression device. The results indicate that dynamic tape without pre-tension does not effectively prevent the immediate reduction in foot arch height after application. Further research is needed to determine the optimal balance between pre-tension and therapeutic efficacy.

## 1. Introduction

The foot arch is a unique and pivotal component of the foot structure [1]. The primary role of the foot arch is to absorb impact following ground contact during routine activities such as walking and running [1]. During shock absorption, the talus pronates, causing the arch height to decrease. By contrast, as the arch length increases, the plantar ligaments and plantar fascia are stretched [2]. This stretching generates a resisting force that counteracts further elongation, thereby helping to stabilize the foot arch [3]. However, when the applied load exceeds the capacity of the foot arch and plantar soft tissues, ailments and disorders related to the foot arch may occur. These disorders are common in clinical practice, such as functional pronated foot, plantar fasciitis, and medial tibial stress syndrome [4,5,6,7,8,9,10].

The key factor in addressing clinical syndromes is supporting the foot arch. When external support is applied to the arch, part of the load is transferred to the supporting structure [11,12], thereby reducing the stress on both the foot arch and the plantar fascia [13,14]. As the load is reduced and kept within the foot arch’s capacity, the injured tissues can rest and heal. Various approaches have been proposed to support the foot arch, such as taping and arch-support insoles [11]. Traditional taping techniques specifically designed to support the foot arch using athletic tape have proven effective in providing substantial support, reducing navicular displacement, and decreasing the pronation of the subtalar and midtarsal joints, thereby alleviating foot arch problems [15,16,17,18,19,20,21]. In addition to athletic tape, various other taping methods, including kinesiology tape, Leukotape, and dynamic tape, are available for physical therapists to support the foot arch. Each type of tape has its own specific techniques developed based on elasticity or stiffness. Dynamic tape offers greater elasticity compared to other types of tape. It is commonly used to restrict motion at the end range of motion of a specific joint. Additionally, dynamic tape has also been reported to reduce pain in foot arch-related disorders, such as plantar fasciitis [22]. However, its mechanical effects on the foot arch remain unclear, particularly under daily cyclic loading. Furthermore, recommendations regarding the pre-tension of dynamic tape during its application remain controversial. Due to the relatively high incidence of skin damage or irritation—reported in up to 20% of cases [23,24]—physical therapists are often reluctant to increase the pre-tension of dynamic tape, which may limit its therapeutic effectiveness. In theory, higher pre-tension provides greater support, but it also increases the risk of skin damage. To minimize the risk of complications, a relatively conservative approach involves applying no tension or only minimal tension; however, the therapeutic effectiveness of this method remains uncertain.

Verifying the effectiveness of interventions is essential for clinical physical therapists, as it helps reduce inappropriate treatments. Therefore, this study aims to examine the initial effects of dynamic taping without pre-tension on maintaining foot arch height by comparing the differences in arch height and arch index between both feet after cyclic loading. One foot was supported with dynamic tape, while the other remained bare for comparison. This study hypothesizes that dynamic tape, when applied without tension, does not effectively support or influence foot arch height.

## 2. Materials and Methods

The present study employed a crossover experimental design, where each participant received the taping intervention on the foot with a relatively low arch height, while the untaped foot served as the control.

### 2.1. Study Subjects

The inclusion criteria for this study were as follows: participants aged 18 or older, with either a normal arch height or physician-diagnosed flatfoot. The participants were not required to possess any specific level of physical fitness. Individuals with a history of lower limb trauma requiring surgical intervention were excluded. A total of thirty-three university students with no history of lower limb trauma voluntarily participated in this study (Table 1). Informed consent was obtained from all participants, and each signed a consent form. For small effect sizes, the number of participants exceeded the minimum sample size suggested in the literature [25].

### 2.2. Taping Technique

**Figure 1:**
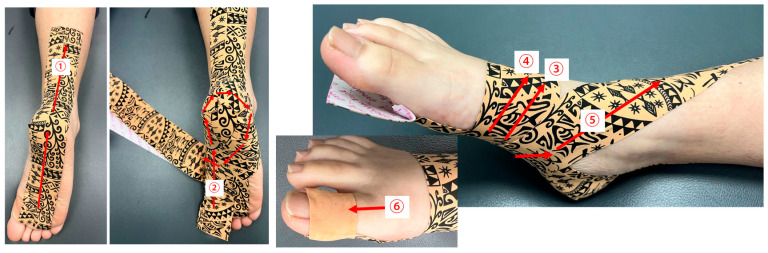
The procedure for applying dynamic tape. The numbers indicate the sequence of movements from smallest to largest.

Dynamic tape (Biomechanical Taping Solutions, Australia), 50 mm wide and 0.1 mm thick, was used to support the foot arch. During its application, subjects were positioned in a prone position with the ankle in maximum plantarflexion and the knee in full extension. In the present study, the arch support taping approach consisted of two procedures (Figure 1). First, longitudinal tape was applied along the sole, extending from the forefoot to the proximal end of the Achilles tendon [19]. Next, an arch support taping technique was performed in one continuous motion, placing the tape along the medial longitudinal arch, wrapping it around the heel from the medial to the lateral side, and then encircling the transverse arch with two strips of tape. Finally, the tape was brought upward through the navicular, creating a supportive sling. During the application, the dynamic tape was gently pulled straight with no additional tension applied.

### 2.3. Apparatus

**Figure 2:**
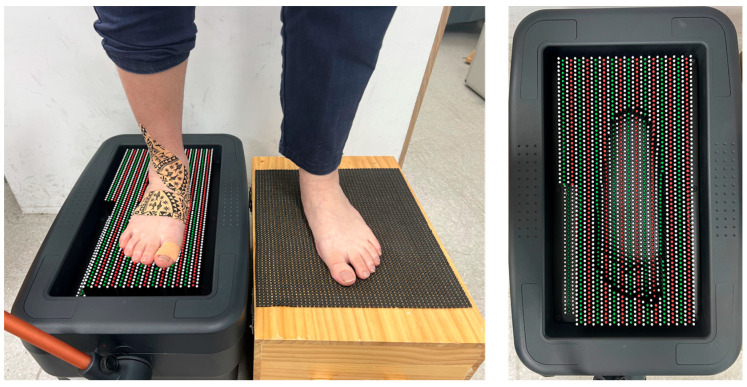
Capturing sole morphology (**left**) and foot impression (**right**).

A commercial foot sole morphology assessment device, the FAST system (Taipei City, Taiwan; http://www.choisfit-taiwan.com, accessed on 15 March 2025), was used to measure the foot arch height with and without dynamic tape after cyclic loading (Figure 2). In the FAST system, a novel single-image-based pin-array impression reconstruction method was used to immediately detect the foot arch height (Figure 2). The device was capable of capturing sole morphology, including foot length, foot width, arch height, and arch index. The FAST system has been validated for intra-rater, inter-rater, and inter-session reliability [26]. Arch height refers to the vertical projection of the measured 3D surface model of the sole. The arch index was calculated as the ratio of the horizontal projection areas of the midfoot and the entire foot. The FAST system has been used to evaluate the sole morphology of cases and the fabrication of arch support insoles.

### 2.4. Test Procedure

**Figure 3:**
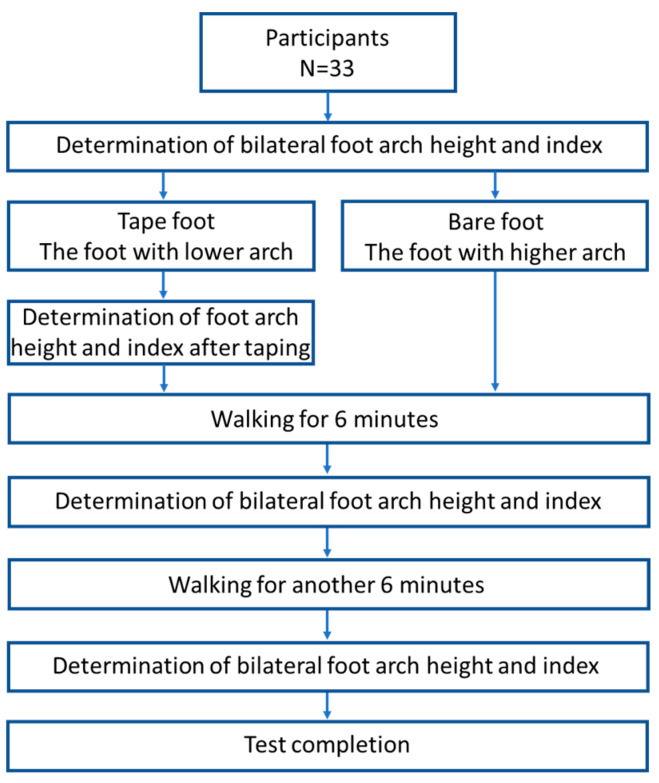
The flowchart of the test procedure.

Participants were required to sit for 30 min to relax their feet before the test. Bilateral foot morphology was assessed to identify the foot with the lower arch height, referring to the side with the relatively lower arch when comparing both feet. Dynamic tape was applied to the foot with the lower arch. The other foot was left untaped to serve as the control condition. The arch height of the taped foot was measured again immediately after taping. Participants were then asked to walk barefoot on a treadmill (RehaWalk, zebris Medical GmbH, Am Galgenbühl, Germany) for 6 min at a self-selected speed that closely resembled their normal walking pace [27]. Bilateral foot morphology was measured again after the 6 min walk. Participants then walked barefoot for another 6 min at a self-selected speed. Finally, bilateral foot morphology was measured again after a total of 12 min of walking (Figure 3).

### 2.5. Index

The foot arch height and arch index obtained from the FAST system were used as indicators to evaluate the effect of the tape in supporting the arch. Foot arch height is defined as the vertical distance between the navicular tubercle and the ground. In addition, the arch index is calculated as the ratio of the contact area of the midfoot to the total contact area of the entire foot on the ground. When the navicular bone lowers under loading, the measured arch height decreases. Consequently, the arch index increases due to the increased contact area between the midfoot and the ground. The foot arch height and arch index have been widely adopted in many studies related to foot arch evaluation. In this study, the values of the arch height and arch index were measured immediately after taping, after 6 min of walking, and after 12 min of walking were compared.

### 2.6. Data Analysis

All statistical analyses were conducted using R software (version 4.4.1). The measurement at 6 min post-taping minus the measurement obtained immediately after taping (before walking) was defined as DiffMin6, whereas the measurement at 12 min post-taping minus the measurement obtained immediately after taping was defined as DiffMin12, thereby quantifying the taping effect at different time points. For each condition, the Wilcoxon signed-rank test was employed to compare the experimental and control sides, with *p* < 0.05 considered statistically significant. Additionally, a Friedman test was performed on the original data at the three time points (“immediately after taping”, “6 min post-taping”, and “12 min post-taping”) to evaluate repeated-measures differences.

### 2.7. Limitations

The device detects sole morphology through direct contact. Therefore, the thickness of the tape was also included in the measurement. Furthermore, the walking trial was limited to a 12 min duration, and walking speeds were not standardized across the participants. These factors introduced inter-individual variability and reduced the ability to control participant-specific differences. In addition, the brief duration may be inadequate for evaluating the potential long-term effects of the intervention. The generalizability of the findings is also constrained by the small sample size, the homogeneous age group, and the predominance of female participants. Therefore, the results should be interpreted with caution, as they may not be representative of the general population.

## 3. Results

The arch height did not decrease significantly after walking for 6 or 12 min on either the taped foot or the untaped foot (Table 2). The mean arch height of the foot with dynamic tape before and after walking for 6 and 12 min was 9.88 ± 3.29 mm, 9.39 ± 3.33 mm, and 9.61 ± 3.43 mm, respectively. Additionally, the mean arch height of the untaped foot was 13.24 ± 4.24 mm before walking, 12.24 ± 4.04 mm after 6 min of walking, and 12.82 ± 4.21 mm after 12 min of walking. However, in female participants, the arch height of the taped foot after 6 min of walking was significantly lower than before walking, decreasing from 9.68 ± 3.43 mm to 8.95 ± 3.37 mm.

The arch index of the taped foot increased significantly from 0.258 ± 0.086 to 0.279 ± 0.072 after 12 min of walking, whereas no significant change was observed in the untaped foot. The arch index of the untaped foot was 0.234 ± 0.081 before walking and 0.240 ± 0.085 and 0.240 ± 0.083 after 6 and 12 min of walking, respectively. No significant differences in arch height or arch index were observed in either the taped or untaped feet after 6 and 12 min of walking compared to baseline (Table 3). The change in arch height of the taped foot after 6 and 12 min of walking was −0.48 ± 1.23 mm and −0.27 ± 1.66 mm, respectively. Similarly, the change in arch height of the untaped foot after 6 and 12 min of walking was −1.00 ± 2.40 mm and −0.42 ± 1.89 mm, respectively.

## 4. Discussion

Dynamic tape, developed based on biomechanical principles, has unique mechanical properties, offering greater elasticity than athletic tape and a higher elastic modulus than kinesiology tape. A pre-tension of more than 50% is required for kinesiology tape to support the foot arch, whereas the application of dynamic tape differs from that of kinesiology tape. When applying dynamic tape, the tension must be carefully controlled, as excessive tension can cause skin damage. Therefore, in the present study, the tape was applied without any pre-stretch tension and simply laid straight on the skin. Inevitably, the supportive and stabilizing effects may be reduced due to the absence of pre-stretch tension. However, evaluating its effectiveness without pre-tension is essential for physical therapists before clinical application.

Dynamic tape has been proposed to relieve pain in cases with plantar fasciitis [22]. The reason behind plantar fasciitis is overloading on the foot arch, and plantar fascia occurs because of the windlass mechanism and foot loading [2,7,28]. Dynamic tape can support the foot arch and share partial loading on the foot arch and plantar fascia. Therefore, the pain is relieved. In the previous study, the dynamic tape was applied to participants for more than 12 h per day, twice a week for 4 weeks. The previous results demonstrated the long-term effect of dynamic tape on the foot arch. Nevertheless, the initial effect is not notable in the present study. The difference in the arch height of the taped foot before and after walking for 6 and 12 min was not notable. The same tendency was observed in the untaped foot. We speculate that the lack of a significant difference in the results may be attributed to the short duration of the testing period. Therefore, a longer walking duration or increased loading may be necessary to adequately assess the effectiveness of dynamic tape.

Athletic tape is traditionally used to support the foot arch, and various taping techniques are proposed, such as anti-pronation, Low-Dye, modified reverse-6, and navicular sling methods [16,20]. Athletic tape has been demonstrated to effectively support the foot arch height by reducing the drop in the navicular bone. However, the primary functions of athletic tape are fixation and protection due to its high stiffness. Almost all motions were limited after the application of athletic tape. Therefore, exercise performance is limited. By contrast, the dynamic tape does not restrict motion until the end range. Hence, dynamic tape is more suitable for sports and exercise. However, the magnitude of pre-tension required for its application necessitates further research.

Kinesiology tape is another option to support the foot arch; however, the major functions of kinesiology tape are lifting and creating space for the fascia and fluid [29]. Furthermore, kinesiology tape is much softer than dynamic tape and athletic tape; therefore, the resistance ability is much lower [30]. Nevertheless, kinesiology tape has recently become popular for the management of many foot disorders, such as function-pronated foot and plantar fasciitis [27,31]. A study comparing taping and extracorporeal shockwave therapy for plantar fasciitis showed no significant difference in their effectiveness [32], highlighting the impact of taping on foot tissues. This suggests that taping techniques using athletic tape, dynamic tape, and kinesiology tape still hold considerable potential for managing various foot-related disorders.

Increasing the magnitude of pre-tension is a widely used technique to enhance the effects of taping, particularly for kinesiology tape. A higher pre-tension has been reported to significantly restrict the ankle complex, whereas kinesiology tape without pre-tension has shown no significant effect [33]. Previous studies have highlighted the effects of pre-tension in the application of kinesiology tape. However, increasing the pre-tension of dynamic tape is more dangerous than that of kinesiology tape due to its higher elastic modulus. Skin damage caused by the high pre-tension of dynamic tape is not uncommon in clinical practice. To minimize the risk of skin damage, physical therapists adopt a conservative approach by applying very low or even no pre-tension. In the present study, dynamic tape was applied without pre-tension because the effectiveness with different pre-tension levels remains unclear. The overall results revealed that foot arch deformation was similar regardless of whether dynamic tape was applied. However, further research is needed to clarify its long-term effects.

In the present study, the dynamic tape was applied to the foot with the lower arch between the bilateral feet while the other foot remained untaped. Directly comparing the differences between bilateral feet is a simple and time-saving approach. However, the disadvantage to this study is that taping was only applied to the weaker foot, making the taping effect on the stronger foot unclear. Nevertheless, in the present study, the arch height of the untaped foot before walking and after 6 and 12 min of walking remained very similar. The results indicate the capacity of the normal foot in bearing loads. Short periods of relaxed walking do not place significant stress on our foot arches, as a normal arch structure is sufficient to bear such a load. Hence, relaxed walking over a short period is a safe exercise suggested for patients with non-structural foot problems.

Age is a critical factor influencing muscle strength and ligament stiffness. In this study, all participants were young university students with no history of foot-related disorders. Their muscles and ligaments were presumed to be in healthy condition, providing sufficient support for their foot arches. Under such circumstances, the benefits of taping may not be readily observable. In contrast, among older adults or individuals with foot disorders, reduced ligament stiffness and muscle strength may increase the likelihood of taping, yielding measurable effects. This highlights the need for further research among different populations. Additionally, the number of female participants exceeded that of males, making the current findings more generalizable to females. Caution should be exercised when interpreting the results for males, given the potential for increased bias due to the smaller sample size.

Detecting foot arch height remains a challenge for researchers, particularly in the context of exercise. Motion-tracking systems with optical markers are commonly used in studies involving high-speed sports due to their precision and accuracy. However, the cost of setting up a motion-tracking system is relatively high, and a large testing area is required to conduct measurements. In contrast, the pin-array impression device used in this study offers a more cost-effective and convenient alternative. It does not require complex setup procedures, making it accessible for various research settings. Nevertheless, a notable disadvantage of the pin-array impression device is the interruption of exercise during measurements. Since the device requires a static condition to capture the foot impression, participants must pause walking and stand still on the device to record the sole impression. This limitation may affect the continuity of the motion assessment during dynamic activities.

Accurately quantifying changes in arch height has long posed a challenge for researchers in the field of foot biomechanics. While direct experimental measurements are commonly used, finite element analysis (FEA) has emerged as a powerful alternative, enabling the evaluation of mechanical responses of the foot arch under various loading conditions. FEA offers several advantages, including the elimination of inter-subject variability and the reduction in measurement-related errors. In addition, it provides comprehensive, full-field data on displacement, deformation, and internal stress throughout the entire model.

Numerous studies have utilized foot finite element models to explore the biomechanical effects of interventions such as taping and footwear, particularly with respect to plantar fascia strain, arch height, and gait [2,34,35]. Simulation results have shown that fascia taping with a high pre-stretch tension of 50% can reduce strain in the plantar fascia and help maintain navicular height [34]. In contrast, conventional Low-Dye taping did not produce significant effects. These findings suggest that high-tension taping may be effective in supporting arch structures. However, such simulations typically do not account for the viscoelastic properties of human soft tissues following cyclic loading. Moreover, the tolerability of high-tension taping by the skin over extended periods remains uncertain and requires further investigation.

Despite its potential, the development of a detailed finite element model that incorporates both anatomical complexity and therapeutic taping interventions remains a significant challenge. The inclusion of complex boundary conditions may lead to difficulties in achieving numerical convergence. Nevertheless, with ongoing advancements in computational power, FEA continues to represent a promising and evolving tool for studying foot biomechanics and optimizing therapeutic strategies.

## 5. Conclusions

Determining the optimal magnitude of pre-tension before applying dynamic tape remains a challenge for physical therapists. While applying dynamic tape without pre-tension minimizes the risk of skin damage, its effectiveness remains uncertain. This study evaluated the immediate effect of dynamic tape applied without pre-tension on foot arch support by directly measuring sole morphology using a pin-array impression device. The quantified foot arch data provide valuable insights for physical therapists. Overall, the results suggest that, in the young adult population, the application of dynamic tape without pre-tension is not recommended for maintaining foot arch height during level walking, at least in terms of its immediate effect. Further research is needed to optimize the balance between pre-tension and therapeutic efficacy.

## Figures and Tables

**Table 1:** The basic data of the participants of this study.

	Participants (N = 33)	Range
Age, years	20.33 ± 0.72	19–22
Body height, cm	164.15 ± 6.66	151–177
Body weight, kg	58.76 ± 12.97	49–100
Rt/Lt	3/32	-
Women/men	22/11	-

**Table 2:** The mean values and standard deviations of arch height (mm) and index in this study.

	Index	Condition	Baseline	6 min	12 min	*p*
Total cases	Arch height (mm)	Taped foot	9.88 ± 3.29	9.39 ± 3.33	9.61 ± 3.43	0.074
		Untaped foot	13.24 ± 4.24	12.24 ± 4.04	12.82 ± 4.21	0.055
		Difference between taped and untaped foot	−3.36 ± 2.68	−2.89 ± 2.22	−3.21 ± 2.41	0.515
	Arch index	Taped foot	0.258 ± 0.086	0.273 ± 0.085	0.279 ± 0.072 *	0.029
		Untaped foot	0.234 ± 0.081	0.240 ± 0.085	0.240 ± 0.083	0.195
		Difference between taped and untaped foot	0.025 ± 0.073	0.034 ± 0.082	0.039 ± 0.083	0.845
Women	Arch height (mm)	Taped foot	9.68 ± 3.43	8.95 ± 3.37 *	9.41 ± 3.54	0.023
		Untaped foot	12.95 ± 4.31	12 ± 3.92	12.64 ± 4.36	0.106
		Difference between taped and untaped foot	−3.27 ± 2.99	−3.05 ± 2.08	−3.23 ± 2.78	0.753
	Arch index	Taped foot	0.254 ± 0.09	0.279 ± 0.087	0.275 ± 0.077	0.133
		Untaped foot	0.225 ± 0.091	0.233 ± 0.089	0.232 ± 0.088	0.071
		Difference between taped and untaped foot	0.029 ± 0.080	0.045 ± 0.090	0.043 ± 0.096	0.919
Men	Arch height (mm)	Taped foot	10.27 ± 3.10	10.27 ± 3.23	10.00 ± 3.32	0.809
		Untaped foot	13.82 ± 4.24	12.73 ± 4.43	13.18 ± 4.07	0.291
		Difference between taped and untaped foot	−3.55 ± 2.02	−2.45 ± 2.54	−3.18 ± 1.54	0.256
	Arch index	Taped foot	0.267 ± 0.080	0.263 ± 0.084	0.285 ± 0.063	0.076
		Untaped foot	0.251 ± 0.055	0.253 ± 0.079	0.255 ± 0.072	0.924
		Difference between taped and untaped foot	0.016 ± 0.057	0.010 ± 0.060	0.030 ± 0.050	0.311

* Significant difference (*p* < 0.05) compared with baseline.

**Table 3:** The mean values and standard deviations of differences in arch height (mm) and index in this study.

	Index	Condition	6 min Later	12 min Later	*p*
Total cases	Difference in arch height (mm)	Taped foot	−0.48 ± 1.23	−0.27 ± 1.66	0.965
		Untaped foot	−1 ± 2.40	−0.42 ± 1.89	0.082
		p	0.289	0.971	
	Arch index	Taped foot	0.015 ± 0.061	0.020 ± 0.062	0.330
		Untaped foot	0.006 ± 0.061	0.006 ± 0.051	0.715
		p	0.543	0.360	
Women	Difference in arch height (mm)	Taped foot	−0.73 ± 1.20	−0.28 ± 1.88	0.472
		Untaped foot	−0.95 ± 2.61	−0.32 ± 2.19	0.172
		p	0.920	0.687	
	Arch index	Taped foot	0.025 ± 0.069	0.021 ± 0.063	1.00
		Untaped foot	0.008 ± 0.065	0.007 ± 0.052	0.911
		p	0.330	0.513	
Men	Difference in arch height (mm)	Taped foot	0 ± 1.18	−0.27 ± 1.19	0.359
		Untaped foot	−1.09 ± 2.02	−0.64 ± 1.12	0.290
		p	0.122	0.257	
	Arch index	Taped foot	−0.005 ± 0.036	0.018 ± 0.064	0.109
		Untaped foot	0.002 ± 0.05	0.005 ± 0.052	0.623
		p	0.759	0.444	

## Data Availability

The data are contained within the article.
